# Can preoperative spino-cranial angle predict cervical sagittal imbalance after laminoplasty?

**DOI:** 10.3389/fnins.2026.1867236

**Published:** 2026-06-19

**Authors:** Yong Li, Peiyan Cheng, Yasen Cao, Xiaolei Zhang, Cai Cheng, Xianzhong Meng

**Affiliations:** 1 Department of Spinal Surgery, The Third Hospital of Hebei Medical University, Shijiazhuang, China; 2 Department of Spinal Surgery, Cangzhou Central Hospital, Cangzhou, China

**Keywords:** cervical sagittal imbalance, cervical sagittal vertical axis, degenerative cervical myelopathy, laminoplasty, spino-cranial angle

## Abstract

**Objective:**

This study aimed to determine whether the preoperative spino-cranial angle (SCA) predicts cervical sagittal imbalance (CSI) after laminoplasty (LP).

**Methods:**

We retrospectively screened 298 consecutive patients with degenerative cervical myelopathy (DCM) who underwent LP at our hospital from January 2018 to June 2021. Of these, 116 met the inclusion criteria and were analyzed. Radiographic and clinical parameters were collected preoperatively and at the last follow-up. Patients were stratified based on the change in cervical sagittal vertical axis (cSVA), calculated as ΔcSVA = postoperative−preoperative, into three categories: improvement (ΔcSVA≤ − 10 mm), stability (−10 mm < ΔcSVA≤10 mm), and deterioration (ΔcSVA>10 mm). Group comparisons were conducted using the *χ*^2^ test, *t*-test, analysis of variance, or non-parametric equivalents, as appropriate. Multivariable logistic regression was used to identify factors associated with postoperative CSI (ΔcSVA>10 mm). Receiver operating characteristic (ROC) analysis was performed to determine the SCA cutoff.

**Results:**

Patients in the deterioration group exhibited the lowest preoperative SCA, along with poorer JOA recovery and worse postoperative neck pain compared to the improvement group. In the adjusted model, a lower SCA independently predicted postoperative cSVA deterioration (OR = 0.575, 95%CI: 0.372–0.808, *p* = 0.012), while other preoperative variables were not significant. The ROC analysis showed fair discrimination (AUC = 0.662), and an SCA threshold of 81.8° yielded 62.6% sensitivity and 96.0% specificity.

**Conclusion:**

The preoperative SCA influences postoperative cervical alignment after LP. Patients with a low SCA are at increased risk for CSI and may benefit from enhanced preoperative counseling and strategies that preserve alignment during LP.

## Introduction

Degenerative cervical myelopathy (DCM) represents a chronic degenerative condition affecting the cervical spine, characterized by spinal cord compression that leads to neural compromise (Bakhsheshian et al., 2017; Nouri et al., 2015). For multilevel disease with cord impingement, laminoplasty (LP) has been broadly adopted as an effective treatment, with generally favorable results reported (Choi and Kang, 2020; Fehlings et al., 2013; Inose et al., 2020). However, the outcomes following LP are not invariably optimal. Patients may experience a loss of cervical lordosis (LCL) and cervical sagittal imbalance (CSI). Previous studies have emphasized that CSI—often indexed by the cervical sagittal vertical axis (cSVA)—is closely linked to postsurgical clinical performance (Sakai et al., 2016; Tang et al., 2015; Zhang et al., 2020). In particular, higher preoperative cSVA was observed to predict a subsequent decline in cervical lordosis and worse recovery after LP (Kim et al., 2020; Zhang et al., 2017). Additional studies have proposed several preoperative radiographic predictors of postoperative CSI, including flexion range of motion (ROM-F), T1-slope (T1s), and the T1s–CL difference (Hyun et al., 2017; Inoue et al., 2021; Lee et al., 2023; Liu et al., 2024). Nonetheless, ROM-F and T1s–CL require paired measurements and calculations. Assessing T1s is frequently challenging because the T1 superior endplate is obscured by the shoulder girdle or rib cage. In addition to its regional biomechanical role, the cervical spine and craniocervical junction are anatomically integrated with the cranial and central neural axes. This anatomical relationship further supports the importance of maintaining cervical sagittal balance (CSB) when planning posterior decompression procedures (Yigit et al., 2024).

The spinocranial angle (SCA) has recently been proposed as a useful metric for assessing CSB (Ling et al., 2018). The SCA, defined as the angle between the C7 superior endplate tangent and a line drawn to the center of the sella turcica (Le Huec et al., 2019) connects the caudal cervical platform to cranial alignment and incorporates head-on-neck gravity, thereby offering a pragmatic alternative when T1-based metrics are difficult to obtain. Huec et al. showed that the SCA is strongly correlated with T1s and CL, with normative values approximating 83° ± 9° (Le Huec et al., 2015). Early evidence also indicates that the SCA is associated with clinical outcomes (Wang et al., 2021) and may forecast LCL (Li et al., 2025). However, the relationship between the SCA and CSI after laminoplasty has seldom been explored.

Accordingly, we conducted a retrospective analysis of patients with DCM to evaluate whether preoperative SCA influences postoperative shifts in cSVA after LP. Our *a priori* hypothesis was that a lower preoperative SCA is associated with a greater likelihood of CSI following LP.

## Methods

### Patient enrollment

**Figure 1:**
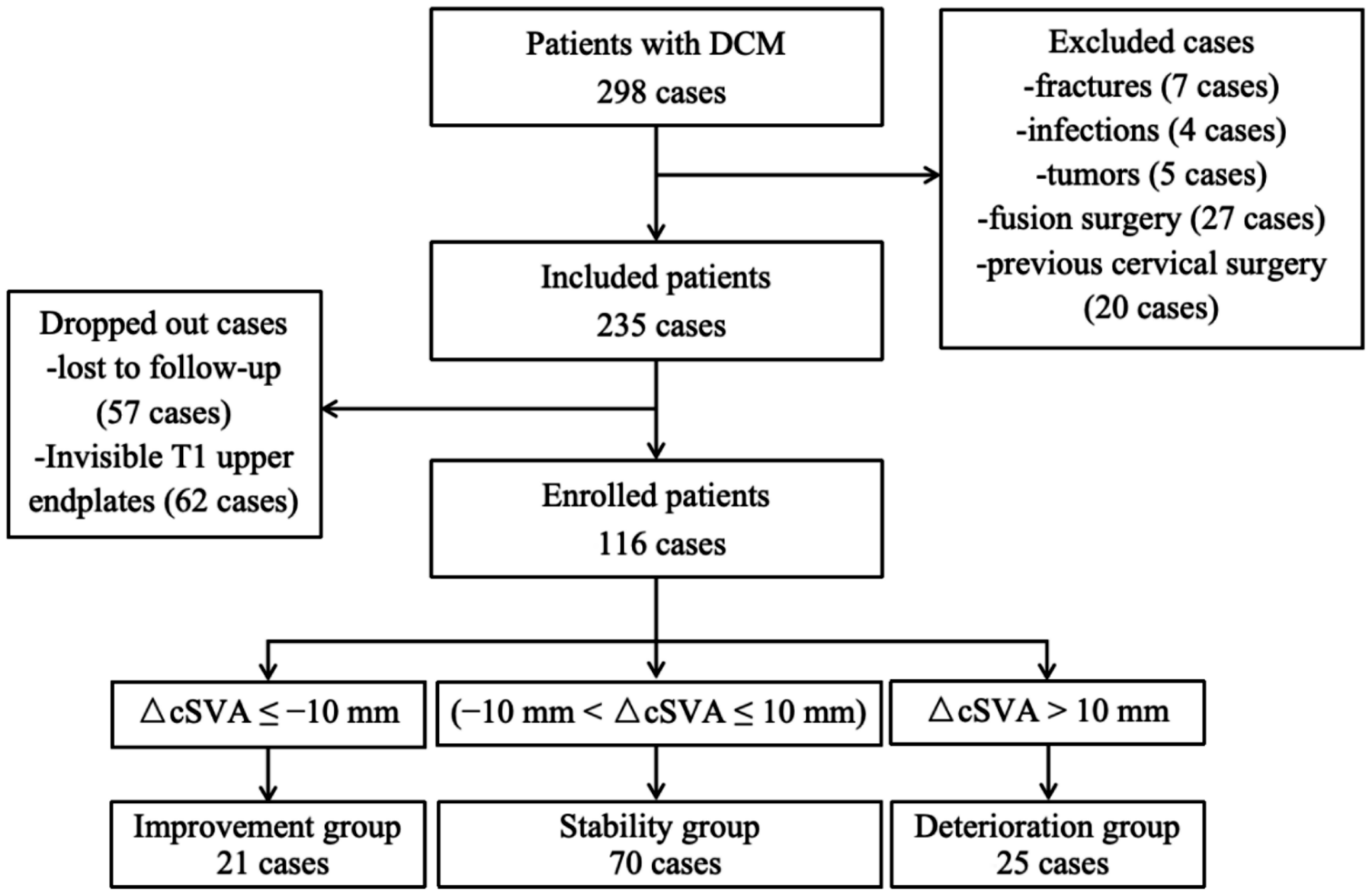
Flowchart of the study.

Approval for this retrospective cohort study was obtained from the institutional review board. Between January 2018 and June 2021, we evaluated a consecutive cohort of 298 individuals who underwent cervical laminoplasty for DCM at a single center. The eligibility criteria were as follows: age ≥18 years, laminoplasty across ≥3 cervical levels, clinical signs consistent with myelopathy, complete radiological records, and a final follow-up of at least 24 months. Patients with a history of cervical operations, tumors, fractures, or infections, those treated in conjunction with cervical fusion, and those whose T1 superior endplate could not be visualized were excluded from the study. All procedures were performed by a high-volume spine surgeon (>200 LP cases annually). The enrollment pathway is summarized in Figure 1.

### Radiologic and clinical parameters

**Figure 2:**
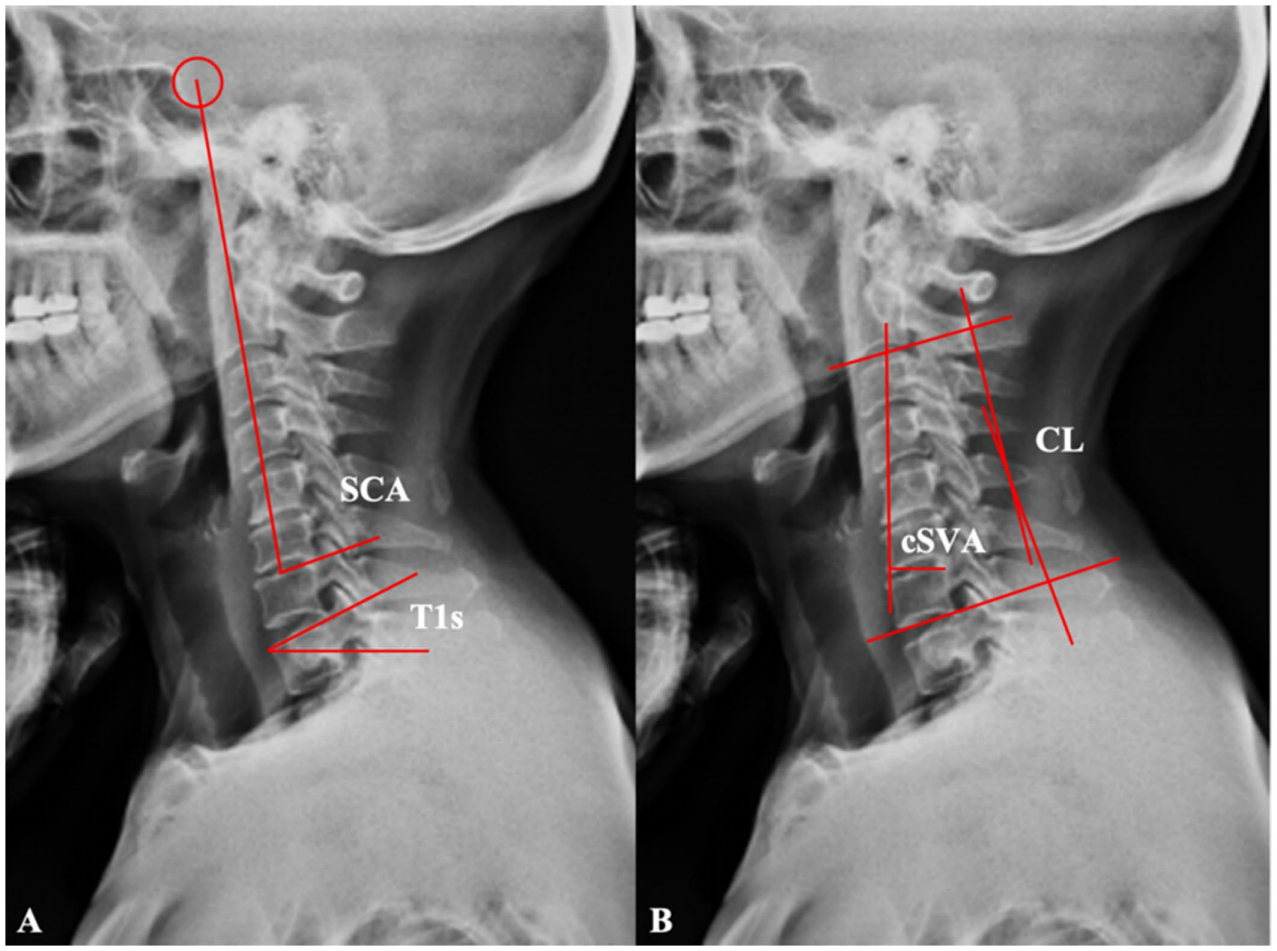
SCA and T1s were measured in the neutral position of **(A)**. CL and cSVA were measured in the neutral position of **(B)**, respectively. SCA indicates spino-cranial angle; T1s, T1-slope; CL, cervical lordosis; cSVA, cervical sagittal vertical axis.

**Figure 3:**
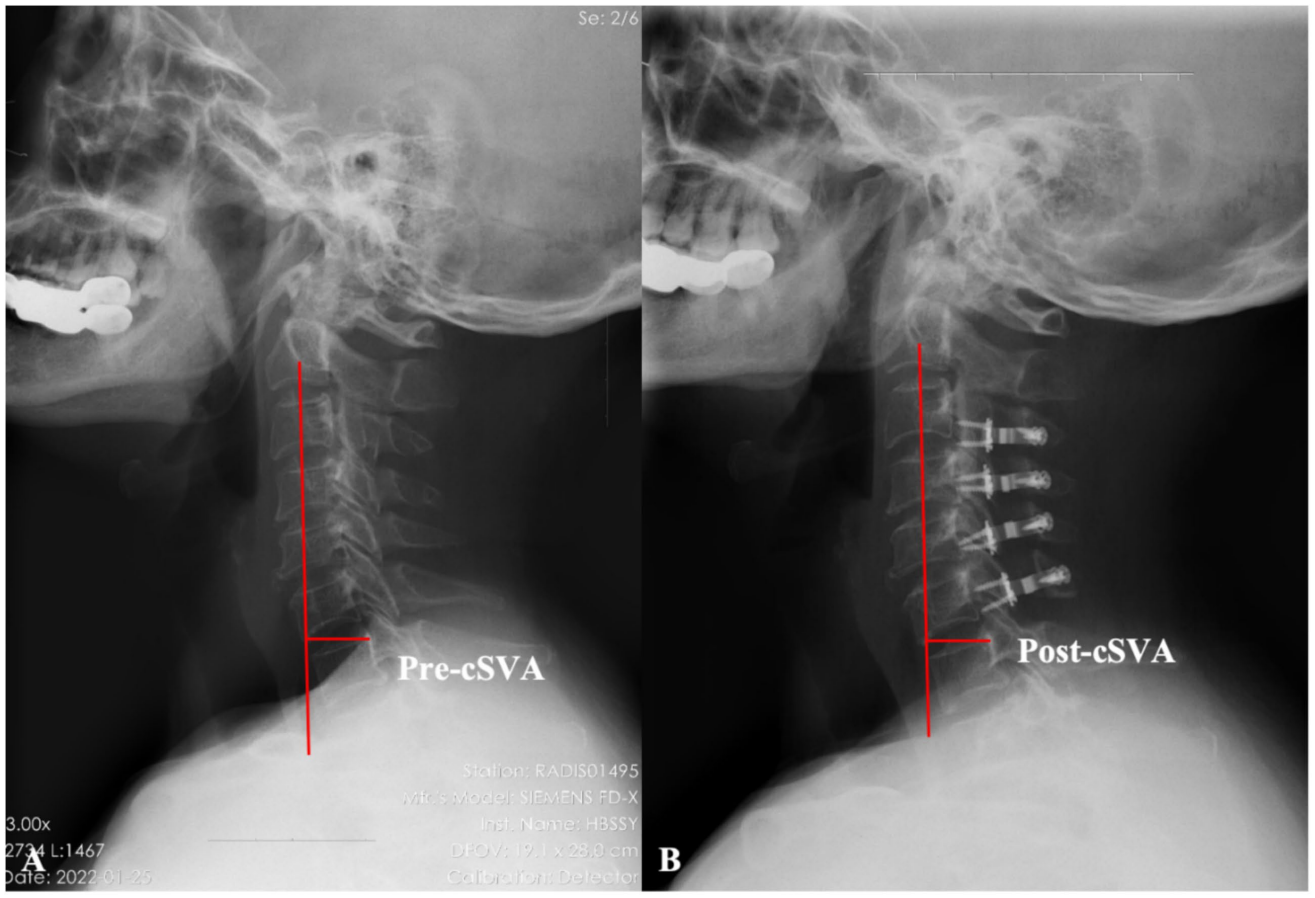
Representative radiographs illustrating the calculation of ΔcSVA. ΔcSVA was defined as postoperative cSVA **(B)** minus preoperative cSVA **(A)**. cSVA, cervical sagittal vertical axis.

Lateral cervical radiographs were used for sagittal measurements (Figure 2). The SCA was defined as the angle formed between the C7 superior endplate tangent and a line drawn from the midpoint of this endplate to the center of the sella turcica. CL was measured between the inferior endplates of C2 and C7. T1s was the angle between a horizontal reference and a line parallel to the T1 superior endplate. The cSVA was measured as the horizontal offset from a vertical plumb line through C2 to the posterosuperior corner of C7. ΔcSVA was defined as the difference between postoperative cSVA and preoperative cSVA (Figure 3). According to previous studies on postoperative cervical sagittal alignment, patients were stratified into three groups: improvement (ΔcSVA ≤ − 10 mm), stability (−10 mm < ΔcSVA ≤ 10 mm), and deterioration (ΔcSVA > 10 mm) (Liu et al., 2024). Neurological recovery was quantified using the Japanese Orthopaedic Association (JOA) score, with the recovery rate calculated as 100 × (postoperative JOA − preoperative JOA)/(17 − preoperative JOA) (Kato et al., 2019). Neck pain intensity was assessed using the Visual Analog Scale (VAS) (Chung et al., 2017).

### Statistical analysis

Data processing was conducted using SPSS 22.0 (SPSS, Inc., Chicago, IL). The distribution of continuous variables was assessed using the Shapiro–Wilk test. As all continuous variables were approximately normally distributed, the data were presented as mean ± standard deviation. Pearson’s correlations were used to evaluate linear associations. Differences between groups for categorical variables were tested using the *χ*^2^ test. Depending on the distributional assumptions, comparisons of radiographic and clinical measures were conducted using either *t*-tests or analysis of variance. Factors related to postoperative CSI were examined using multivariable logistic regression. Receiver operating characteristic (ROC) analysis was performed to identify optimal thresholds. Two-sided tests were performed, and statistical significance was defined as a *p*-value of <0.05.

## Results

### Patient characteristics

A total of 116 patients were analyzed, consisting of 78 men (67.2%). The mean BMI was 26.7 ± 4.3 kg/m^2^. Neurological function improved from a preoperative JOA score of 11.5 ± 1.6 to 15.1 ± 1.5 at the last follow-up, with a mean JOA recovery rate of 65.2 ± 28.5%. Pain levels decreased from a preoperative VAS score of 3.0 ± 1.6 to 2.1 ± 1.5. The majority of procedures involved the fusion of 3–4 levels, with 3 levels fused in 51 patients (44.0%), 4 levels in 44 patients (37.9%), and 5 levels in 21 patients (18.1%). The proximal instrumented level was C3 in 57.8% of cases (67/116). The mean follow-up duration was 28.3 ± 6.2 months (Table 1).

**Table 1:** Patient population.

Demographic	Value
Sex, *n* (%)	
Male	78 (67.2%)
Female	38 (32.8%)
BMI, kg/m^2^	26.7 ± 4.3
Pre-JOA	11.5 ± 1.6
Post-JOA	15.1 ± 1.5
JOA recovery rate (%)	65.2 ± 28.5
Pre-VAS	3.0 ± 1.6
Post-VAS	2.1 ± 1.5
Surgery segment, *n* (%)	
3 levels	51(44.0%)
4 levels	44(37.9%)
5 levels	21(18.1%)
Proximal level, *n* (%)	
C3	67(57.8%)
C4	49(42.2%)

BMI, body mass index; JOA, Japanese Orthopedic Association; VAS, visual analog scale.

### Correlations analysis between ΔcSVA and preoperative factors

Pearson analysis showed that older age (*r* = 0.275), higher preoperative T1s (*r* = 0.253), and greater preoperative CL (*r* = 0.217) correlated with a larger forward shift in cSVA postoperatively. In contrast, larger preoperative SCA (*r* = −0.327) and larger preoperative cSVA (*r* = −0.251) correlated with a smaller forward shift (all *p* < 0.05). The T1s–CL mismatch was not significantly associated with ΔcSVA (*r* = −0.106) (Table 2).

**Table 2:** Correlations analysis between ΔcSVA and preoperative factors.

Parameters	Mean ± SD	Pearson	*p*
Age, years	68.6 ± 11.2	0.275	0.003
Follow-up period, months	28.3 ± 6.2	0.144	0.123
Pre-SCA, °	82.8 ± 6.9	−0.327	<0.001
Pre-CL, °	17.4 ± 8.5	0.217	0.019
Pre-T1s, °	27.7 ± 7.6	0.253	0.006
Pre-cSVA, mm	27.7 ± 10.2	−0.251	0.007
Pre-T1s − CL, °	10.3 ± 8.1	−0.106	0.257

SCA, spino-cranial angle; CL, cervical lordosis; cSVA, cervical sagittal vertical axis; T1s, T1 slope.

### Comparison of clinical outcomes based on postoperative ΔcSVA

Patients were stratified as improvement (ΔcSVA≤ − 10 mm; *n* = 21), stability (−10 mm < ΔcSVA≤10 mm; *n* = 70), and deterioration (ΔcSVA>10 mm; *n* = 25) (Table 3). All three groups experienced significant within-group gains in JOA (all *p* < 0.05). Between groups, the improvement cohort achieved the highest JOA recovery rate (77.4 ± 31.3%), whereas the deterioration cohort had the lowest (46.9 ± 30.4%; *p* < 0.05). For pain, the improvement group had the lowest postoperative VAS (1.6 ± 1.2) and the deterioration group the highest (2.8 ± 1.4; *p* < 0.05); notably, pain reduction did not reach significance in the deterioration cohort.

**Table 3:** Comparison of clinical outcomes based on postoperative ΔcSVA.

Parameters	Improvement (*n* = 21)	Stability (*n* = 70)	Deterioration (*n* = 25)	Between-group *p*-value
Pre-JOA	10.8 ± 2.6	11.1 ± 1.5	12.1 ± 1.5	0.083
Post-JOA	15.6 ± 1.3	15.2 ± 1.9	14.4 ± 1.4	0.148
Within-group *p*-value	<0.001	<0.001	<0.001	—
JOA recovery rate (%)	77.4 ± 31.3^†^	69.5 ± 32.7	46.9 ± 30.4^†^	<0.001
Pre-VAS	3.3 ± 2.2	3.0 ± 1.8	2.7 ± 1.7	0.451
Post-VAS	1.6 ± 1.2^†^	2.1 ± 1.5	2.8 ± 1.4^†^	0.018
Within-group *p*-value	<0.001	<0.001	0.753	—

^†^indicates a significant pairwise difference between the improvement and deterioration groups.

Improvement group, ΔcSVA ≤ − 10 mm; stable group, −10 mm < ΔcSVA ≤ 10 mm; deterioration group, ΔcSVA > 10 mm; JOA, Japanese Orthopedic Association; VAS, visual analog scale.

### Comparison of evaluated parameters based on postoperative ΔcSVA

Groupwise comparisons were directionally consistent with the correlation findings (Table 4). The improvement group had the highest preoperative SCA (86.1 ± 5.6°), whereas the deterioration group had the lowest (79.8 ± 7.1°; *p* < 0.05). Conversely, preoperative CL and T1S were greatest in the deterioration group (20.1 ± 9.8° and 28.9 ± 6.6°, respectively; both *p* < 0.05). Preoperative cSVA was the largest in the improvement cohort (35.1 ± 10.5 mm), intermediate in the stability cohort (27.5 ± 10.8 mm), and smallest in the deterioration cohort (21.3 ± 11.1 mm; *p* < 0.05). The proportion of constructs with a proximal level at C3 increased stepwise across strata (42.9% to 54.3% to 80.0%; *p* < 0.05). Fused-level counts did not differ significantly among groups.

**Table 4:** Comparison of evaluated parameters based on postoperative ΔcSVA.

Parameters	Improvement (*n* = 21)	Stability (*n* = 70)	Deterioration (*n* = 25)	*p*
Age, years	65.6 ± 8.9	68.1 ± 9.5	70.7 ± 11.6	0.121
Sex, *n* (%)				0.509
Male	13 (61.9%)	50 (71.4%)	15 (60.0%)	
Female	8 (38.1%)	20 (28.6%)	10 (40.0%)	
BMI, kg/m^2^	25.4 ± 4.3	26.6 ± 4.1	27.2 ± 4.9	0.274
Follow-up period, months	27.1 ± 6.8	28.5 ± 5.3	28.9 ± 7.2	0.563
Surgery segment, levels	3.68 ± 0.8	3.8 ± 0.8	3.7 ± 0.7	0.760
Proximal level (C3)	9(42.9%)	38(54.3%)	20(80%)	0.043
Pre-SCA, °	86.1 ± 5.6^†^	83.0 ± 5.1	79.8 ± 7.1^†^	0.001
Pre-CL, °	15.1 ± 7.5^†^	17.2 ± 6.9	20.1 ± 9.8^†^	0.027
Pre-T1s, °	24.7 ± 6.1^†^	27.0 ± 7.4	28.9 ± 6.6^†^	0.034
Pre-T1s − CL, °	9.6 ± 7.2	9.9 ± 6.8	8.8 ± 8.1	0.605
Pre-cSVA, mm	35.1 ± 10.5^†^	27.5 ± 10.8^†^	21.3 ± 11.1^†^	<0.001

^†^Indicates *p* ≤ 0.05 between two groups.

Improvement group, ΔcSVA ≤ − 10 mm; stable group, −10 mm < ΔcSVA ≤ 10 mm; deterioration group, ΔcSVA > 10 mm; SCA, spino-cranial angle; CL, cervical lordosis; cSVA, cervical sagittal vertical axis; T1s, T1 slope.

### Risk factors for postoperative deterioration of cSVA

**Figure 4:**
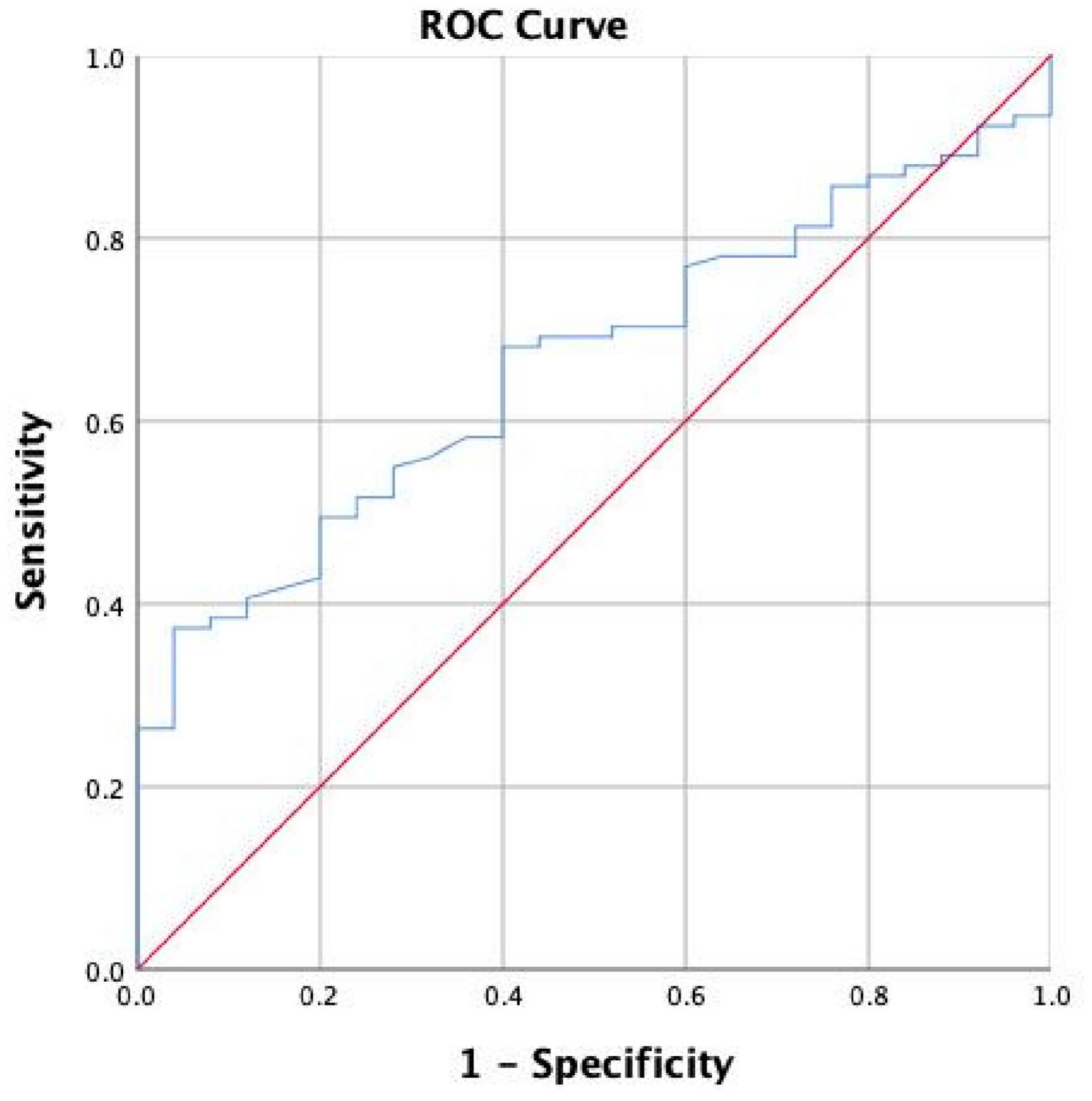
ROC curve analysis to predict △cSVA >10 mm (AUC = 0.662, *p* = 0.013). The cutoff value for SCA was 81.8°, with a sensitivity of 62.6% and a specificity of 96.0%. AUC indicates area under the curve; △cSVA, changes of the cervical sagittal vertical axis; ROC, receiver operating characteristic; SCA, spino-cranial angle.

The multivariable model included proximal surgical level, preoperative SCA, preoperative CL, preoperative T1s, and preoperative cSVA because these variables were clinically relevant to postoperative sagittal alignment and showed potential associations in univariate or groupwise analyses. On multivariable logistic regression (Table 5), a larger preoperative SCA independently protected against postoperative cSVA deterioration (OR = 0.575; 95%CI, 0.372–0.808; *p* = 0.012). A proximal instrumented level at C3 showed a borderline association with deterioration risk (OR = 5.481; *p* = 0.089). Preoperative CL, T1S, and cSVA were not independent predictors in the adjusted model. ROC curve revealed that preoperative SCA demonstrated fair discrimination for identifying patients at risk of ΔcSVA>10 mm (AUC = 0.662; *p* = 0.013) (Figure 4). A cutoff value of 81.8° yielded a sensitivity of 62.6% and a specificity of 96.0%, supporting the SCA as a high-specificity “rule-in” indicator for forward shift risk.

**Table 5:** Risk factors for postoperative deterioration of cSVA (ΔcSVA>10 mm).

Variable	*p*	OR	95% CI
Proximal level (C3)	0.089	5.481	0.667–13.898
Pre-SCA (°)	0.012	0.575	0.372–0.808
Pre-CL (°)	0.473	0.885	0.342–1.596
Pre-T1s (°)	0.370	0.935	0.626–1.678
Pre-cSVA (mm)	0.163	0.911	0.896–1.019

cSVA, cervical sagittal vertical axis; OR, odds ratio; CI: confidence interval; SCA, spino-cranial angle; CL, cervical lordosis; T1s, T1 slope.

## Discussion

This study examined whether preoperative SCA was associated with ΔcSVA and identified risk factors for CSI after LP. We found a negative association between preoperative SCA and ΔcSVA, indicating that a lower SCA is linked to a greater forward shift of the head relative to C7. In multivariable analysis, a lower SCA was identified as a risk factor for postoperative cSVA deterioration, and an SCA threshold of 81.8° effectively distinguished patients at higher risk. These data support the incorporation of SCA into preoperative planning for LP.

CSB is integral to maintaining a neutral head position and a horizontal gaze (Tang et al., 2019). When CSB is disturbed, compensatory overwork of the posterior cervical musculature contributes to axial neck pain (Kim et al., 2020; Pinter et al., 2023). CSI often coexists with LCL and kyphotic shift, which may limit the dorsal migration of the cord following LP, thereby compromising decompression efficacy (Zhang et al., 2020; Kim et al., 2021). Previous reports have consistently linked cSVA-based imbalance with worse symptoms and functional outcomes (Chen et al., 2020; Koshimizu et al., 2018; Oshima et al., 2016; Roguski et al., 2014; Xu et al., 2020). In line with those observations, our cohort’s deterioration group exhibited inferior JOA recovery than the stability and improvement groups. Marked postoperative increases in cSVA were associated with greater LCL and reduced space for posterior cord drift, plausibly explaining suboptimal neurological recovery (Machino et al., 2020; Pan et al., 2020). Larger cSVA has also been associated with higher intramedullary pressure and histological cord changes, offering a biological substrate for clinical decline (Shimizu et al., 2005; Winestone et al., 2012). Consistently, patients with larger pre- and postoperative cSVA in our series reported higher levels of neck pain, with improvements confined to those in the improvement groups—an observation that aligns with prior literature (Liu et al., 2024) and is compatible with excessive suboccipital muscle contraction as a pain generator (Fakhran et al., 2016; Patwardhan et al., 2018).

Multiple sagittal parameters correlate with cSVA; typically, cSVA correlates positively with T1s, T1s–CL, and C2 slope and negatively with CL (Inoue et al., 2021; Rao et al., 2019; Weng et al., 2016). Previous studies suggested that lower preoperative SCA associates with higher postoperative cSVA after LP (Wang et al., 2021), and several studies have also highlighted strong relationships between SCA and other sagittal indices, as well as clinical outcomes after ACDF and LP—particularly where T1 visualization is limited, and SCA can act as a proxy for T1-based measures (Liu et al., 2022; Wang et al., 2021; Wang et al., 2021). In contrast to LP, multilevel ACDF may directly reconstruct disc height and segmental lordosis, thereby modifying postoperative cervical alignment through a different biomechanical pathway. However, ACDF also alters motion distribution and may influence adjacent-segment mechanics. Therefore, the alignment changes observed after LP should not be viewed as a general consequence of cervical surgery alone but rather as an LP-specific phenomenon related to decompression without fusion, partial disruption of posterior stabilizing structures, and dependence on residual extensor muscle function (Gokoglu et al., 2025). Few investigations have specifically addressed SCA’s impact on ΔcSVA after LP; our data help fill that gap by demonstrating an inverse SCA–ΔcSVA relationship and by quantifying a clinically actionable cutoff.

SCA integrates the cranial center of mass with the cervical base, capturing head-on-neck alignment in a single measure. The following complementary mechanisms may explain why lower SCA predisposes to postoperative CSI: To preserve horizontal gaze, patients with lower SCA must rely on greater tonic activity of the cervical extensors even at baseline. After LP—where spinous processes, interspinous ligaments, and posterior tension-band integrity are partially sacrificed—the system loses part of its passive stabilizing restraint, leaving insufficient “reserve” to counter head-forward moments. In addition, by removing posterior elements, LP shifts reliance from passive structures to muscular control. In low-SCA spines, the extensor moment arm is disadvantaged, raising the energetic cost of maintaining head position. Fatigability and micro-failure of posterior soft tissues can promote progressive anterior drift and LCL—manifesting clinically as CSI and axial pain. The importance of preserving posterior craniospinal anatomy is also supported by evidence from other posterior craniocervical procedures. In surgeries involving the craniocervical junction, such as Chiari malformation decompression, excessive disruption of posterior osseoligamentous and muscle-tendon structures may affect regional stability and postoperative alignment (Yigit and Gokoglu, 2025). This concept is consistent with the present findings, because SCA geometrically links the cranial reference point to the lower cervical base and may therefore reflect the functional interaction between head position, craniocervical anatomy, and lower cervical sagittal alignment.

Although the SCA cutoff of 81.8° showed high specificity, the overall discriminatory performance was modest, with an AUC of 0.662. Therefore, SCA should not be regarded as a standalone predictive tool for postoperative sagittal deterioration. Rather, it may be considered an adjunctive radiographic marker that helps identify patients who warrant closer preoperative assessment, more careful preservation of posterior stabilizers, and closer postoperative radiographic follow-up. Mechanistically, patients with SCA > 81.8° may require less compensatory extensor effort to maintain horizontal gaze, making them more resilient to the posterior tension-band attenuation inherent to LP. In such patients, surgeons should anticipate a greater alignment penalty after LP and prioritize preservation of posterior stabilizers (meticulous handling of the interspinous–supraspinous complex and muscle insertions). Preoperative counseling should include the potential for postoperative forward shift and axial pain in low-SCA individuals, and closer radiographic follow-up may be warranted.

Several limitations merit consideration. First, the retrospective, non-randomized design introduces potential selection bias. Second, we did not quantify paraspinal muscle quality or fatty infiltration, which may moderate the SCA–CSI relationship. Third, the ROC performance was modest, and the number of patients with postoperative cSVA deterioration was limited. These factors reduce the statistical power and precision of the cutoff estimate. The proposed threshold should therefore be interpreted as exploratory and hypothesis-generating. Future multicenter prospective studies with larger sample sizes and external validation cohorts are needed before this cutoff can be applied as a robust clinical decision-making threshold. Finally, although our minimum follow-up was 24 months, longer surveillance could refine the natural history of alignment change; that said, prior studies suggest that post-LP sagittal changes plateau within 6 months (Choi et al., 2018), supporting the adequacy of our follow-up for the primary endpoints.

## Conclusion

Preoperative lower SCA independently associates with a higher ΔcSVA and higher risk of CSI after LP. Beyond its practicality when T1 is obscured, SCA encapsulates head–neck biomechanics in a single angle, offering a mechanistically plausible and clinically actionable predictor. Incorporating SCA into preoperative assessment may improve patient selection, surgical planning, and postoperative counseling.

## Data Availability

The raw data supporting the conclusions of this article will be made available by the authors, without undue reservation.
